# Does Public Service Motivation Affect Teacher Satisfaction From the Perspective of Urban and Rural Dual Structure? Empirical Analysis Based on Estonia TALIS 2018 Data

**DOI:** 10.3389/fpsyt.2022.727659

**Published:** 2022-05-27

**Authors:** Xiaodi Jiang

**Affiliations:** Institute of International and Comparative Education, Beijing Normal University, Beijing, China

**Keywords:** urban-rural dual structure, public service motivation, Estonia, TALIS, teacher satisfaction

## Abstract

In recent years, basic education in Estonia has achieved balanced development despite the imbalance of structure and regional factors, and this phenomenon has made the Estonian educational system a general interest of international scholars. Based on the data from the 2018 Teacher Teaching International Survey (TALIS) database, this research divides 3,004 respondents in Estonia into rural teachers and urban teachers and explores the impact of public service motivation on teacher satisfaction from the perspective of urban-rural dual structure through the grouping and comparison of ordered regression models. The study finds that the three dimensions of public service motivations in Estonia will positively affect teacher satisfaction and that the influence coefficient of contribute to the society and the tendency to participate in education policies on teacher satisfaction is higher in rural areas than in urban areas. In addition, the satisfaction of rural teachers in Estonia will decrease with the age of teachers, and the satisfaction of male teachers will be significantly lower than the satisfaction of female teachers, while the satisfaction of urban teachers will decrease with the improvement of academic qualifications. In terms of the implications, on the one hand, Estonia's education policy attempts to solve the imbalance between the regional and teacher structure through subsidies and incentives; on the other hand, Estonia's education policy uses measures such as multidimensional evaluation, multichannel feedback, and local democratization to increase the teacher's participation as well as their satisfaction.

## Introduction

In the past two decades, the achievements of the Finnish education model have attracted global attention because, in addition to factors such as economy and system, the lofty status of teachers in national culture, the high degree of autonomy in teacher work, and the people's enthusiasm for public service have created a high-quality and efficient teacher group (1).

The “Finnish Miracle” has also aroused academic thinking about teacher satisfaction, which has gradually formed an exploration of “non-salary driving satisfaction factors” in comparative education research. The 2018 PISA (the Program for International Student Assessment) report witnessed Estonia's tremendous achievement in terms of basic education, which replaced Finland's place of highest average score of all OECD countries. Estonia, a country with only 1.3 million people, restored its sovereignty in 1991. In terms of endowment factors in Estonia, scholars tend to explain educational improvement through rapid economic development steps (2), historical path dependence of public schooling (3), homogeneity of family and well-educated parents and great social recognition of basic education. One the scope of educational system, comprehensive school principals, output-oriented curricula, modern textbooks and new assessments of education outcomes could partly express Estonia's remarkable achievement in basic education (4). Compared with the factors mentioned above, the issue of teachers in Estonia and the environment in which they work is neglected by most research, which leaves a gap in Estonian educational research.

Both the countries of the former Soviet Union and most emerging countries are facing the problem of the dual structure of urban and rural areas. The education gap caused by uneven regional development is a major obstacle to the equalization of public services and the efficiency of supply. As a matter of fact, Estonia has a tendency of lock-in: The first issue is the poverty of the education sector, and education expenditure in the country's GDP is far below the average level of OECD countries. The second issue is the general aging of the teaching staff. Nearly half of Estonian teachers held teacher positions before the disintegration of the Soviet Union, and the gender ratio is seriously imbalanced (more than 80% of teachers are female). The last issue but not the least, the salary of teachers is significantly lower than the salaries of other professions in Estonia (equivalent to 68% of the domestic salary level) and the OECD national average (teacher salary accounts for only 55% of the average teacher salary of OECD countries), and the welfare of teachers is far lower than in other developed countries (5). However, Estonia has made remarkable achievements under the condition of weak endowment. According to the analytical framework of comparative education, the education performance of a country is often determined by the educational environment (including factors such as national cultural characteristics, globalization level, immigration policy, leadership, and degree of democracy) and educational devotion (6). As the most important participant in basic education, teacher satisfaction, cognitive ability, and salary are important indicators that determine the regional education level. According to the results of the international survey of teacher teaching, the average satisfaction of teachers in Estonia is much higher than the average satisfaction of teachers in OECD countries. Moreover, there is no difference in satisfaction due to the difference between the urban and rural education environment and other educational elements (7). Estonia's high teacher satisfaction level is undoubtedly a strong driving force for the development of basic education. Exploring the source of teacher satisfaction could provide experience for education reform in emerging countries during the transition period and find a model and path for underdeveloped and rural schools to get out of the “floating trend” predicament.

Due to the existence of the dual structure of urban and rural areas, Estonia has also encountered problems such as the lagging development of rural education facilities, the widening of the urban-rural gap in teacher treatment, and the obstruction of the promotion route of rural teachers. Therefore, although Estonia has implemented a local autonomous school governance model and delegated power to local schools after independence, problems such as the misplacement of teacher education supply and demand, low teacher salaries, and fewer opportunities for professional development of rural teachers are difficult to solve in the short term. In this way, Estonian education policy decision makers and local school principals have very limited ways and means to motivate rural teachers. Therefore, in policy formulation and educational strategic development planning, Estonia has to pay more attention to the shaping and utilization of cultural concepts (8). Recognizing that attitude determines teacher behavior and behavior determines teacher efficiency, the theory of public service motivation is used in research to explore the source of teacher satisfaction. Public service motivation is a concept put forward by the upper world in the 1990's. Based on being aware of the limitations of public choice theory, specifically for public employees, the public sector should encourage public service providers through value selection and guidance, social control, and self-concept construction (9). Public service motivation includes multiple dimensions, such as geo-culture, values, and sense of belonging, all of which have an impact on public service participants, and higher public service motivation can often lead to higher public service efficiency (10). Compared with other public services, the main utility of education as a public service in the implementation process comes from the satisfaction of teachers, and the impact of this element is much higher than the impact of other elements (such as capital, technology, and systems). Hence, studying the influence of public service motivation on teacher behavior, teacher satisfaction, and teacher performance has gradually become an important topic. Similar to neighboring Finland, Estonia has a happy and dedicated team of teachers. In addition, despite the large difference between urban and rural education environments and teacher treatment, rural teachers still maintain a high degree of satisfaction (11). Does the public service motivation of teachers play a more important role in rural areas in Estonia? To solve this problem could not only explain the internal mechanism of the transition of education but also provide implications for emerging countries to improve the level of education elements and the quality of education^1^.

There are two main research questions in this study: one is to explore the influence of public service motivation in Estonia on teacher satisfaction, and the other is to compare the effects of public service motivation between urban teacher and rural teacher. In this way, the author intends to combine the database data of the Teacher Teaching International Survey (TALIS) to divide teachers in Estonia into rural and urban areas according to their schools and to verify the utility of public service motivation and the influence of the urban-rural dual structure through quantitative analysis. The rest of the paper includes a summary of related theories and research and hypotheses, research methodology, variable design and model establishment, and the main findings and discussions of the research. In the final section, the author will rethink Estonia's educational reform about the research findings and summarize the shortcomings and deficiencies of the research with the aims of looking forward to the development direction of future research.

## Literature Review

### The Theory of Urban and Rural Dual Structure and Its Influence

The urban-rural dual structure is also called the “Lewis-Fei-Ranis Model,” and it was developed from Lewis's views on the dynamic connection between rural and urban industries (12). The urban-rural dual structure itself is an economic phenomenon that refers to a special form of a serious imbalance between the urban and rural industrial structure and the distribution of production factors in developing countries. Specifically, cities are dominated by vertically integrated capital and intellectually and technologically capital-intensive industries, while rural areas are dominated by individual production. Therefore, urban infrastructure and cultural soft environments are relatively developed, while rural areas are relatively backward (13). The dual structure of urban and rural areas has been an inevitable problem in the modernization process of emerging countries since the middle and late twentieth centuries. Since late-developing countries cannot achieve primitive accumulation through colonization and international trade monopoly like early capitalist countries, they can achieve the rapid development of urbanization and industry by extracting surplus value from agriculture to obtain the primitive accumulation required for industrialization development, which became the early Soviet planned economic system. This approach has led to the dilemma of “low-end lock-in” in rural development (14), which has also led to many social problems, especially education problems. On the one hand, the harsh environment in rural areas affects the accessibility of education. On the other hand, economic problems are magnified, education investment is unbalanced between urban and rural areas, and the individual needs of teachers and students in rural areas cannot be met, which has led to the urban-rural duality of education quality and education equity. In education issues, equity and efficiency are often interrelated and mutually reinforcing. Under the dual structure of urban and rural areas, rural schools are facing problems such as a shortage of teacher resources, aging of the teaching staff, and structural shortages (15). Compared with urban teachers, rural teachers face problems such as a single performance appraisal, fewer promotion channels, low social recognition, poor living conditions and a poor educational environment for children. These problems have reduced the satisfaction and willingness of rural teachers, which has further widened the education gap between rural and urban areas (16).

Baltic states such as Estonia had joined the Soviet Union as member countries for more than 70 years, and their political, economic and social development has been assimilated to a large extent. Although long-term institutional, economic, and educational reforms were carried out after independence in the 1990's, the problems are still more significant because the dual structure of urban and rural areas has gone through three stages: formation, development, and solidification. In past studies, rural teachers in Estonia faced more problems with less compensation and fewer opportunities (17). The problems brought about by the urban-rural dual structure greatly affect the satisfaction of teachers. Meanwhile, some scholars have explained the duality of urban-rural education from another perspective. Compared with rural teachers, urban teachers have more satisfactory salaries, more training opportunities, a more transparent promotion model and a better working environment. Since all factors are kept at a relatively high level, increasing the level of factors has a small impact on teacher satisfaction and even affects the positive effects of other factors on teacher satisfaction, forming a crowding-out effect. In contrast, raising the level of a single factor has a relatively high marginal effect on rural teacher satisfaction. Therefore, in the case of relatively low environmental factors and salary factors, social recognition of teachers, regional culture, and sense of dedication will all have a greater impact on teacher satisfaction (18, 19).

### Public Service Motivation

In 1990, the famous public management scholar Perry put forward the concept of public service motivation and gave a clear connotation and internal dimensions. Integrating the origin and background of public service motivation, it is not difficult to find that public service motivation is the product of the “New Public Management Era.” Early researchers found that people serving in the public sector tend to participate in meaningful public services more than private and individual sector workers. These intrinsic motivations, on the one hand, maintain a sense of gain in work when public servants are relatively low in salary, working environment, and social status (20). On the other hand, public service motivation also dives the labor force to lean toward the public service sector when choosing an occupation. According to Perry's definition, public service motivation is divided into four dimensions, including the commitment to participate in public service, participation in public policy, compassion, and sense of self-sacrifice. The proposal of public service motivation, along with the discussion of incentives in the “New Public Management Era,” has spawned more empirical studies, including research on causal factors (personality characteristics, regional culture, organizational structure) and outcome research (work attitude, performance, career choice and willingness to leave). Among these factors, empirical research and experimental research are the main research methods (21). With the further in-depth study of public service motivation and the globalization of research objects, comparative studies and meta-analyses have become important research methods for public service motivation research. For instance, Homberg et al. (22) applied a meta-analysis to analyze the influence of public service motivation on job satisfaction. As a statistically integrated methodology, meta-analysis has played an important role in the extension of public service motivation to other disciplines based on the summary and classification of previous studies. Comparative research can compare the public service motivation and influencing factors of different types of research objects in terms of region, culture, economic development level, industry nature, etc. On the one hand, the purpose of comparative research is to extend the theoretical study of public service motivation and to form countermeasures and suggestions that are conducive to improving public service motivation. In recent years, public service motivation's research areas have expanded to developing countries. Yudiatmaja (23) proved that public service motivation could positively affect public servant's job satisfaction. In China, public service motivation is found to positively affect officer's wellbeing as well as moderates the relationship between work stress and satisfaction.

There are two main reasons why educational studies pay attention to the theory of public service motivation: one is that the academic community has discovered that principals can “awaken” teachers' public service motivation, similar to leaders in the public system. The other reason is that teachers with high public service motivation can often invest more in their work, and the degree of teacher engagement can often lead to better academic performance of students (24). In terms of whether public service motivation could act on teacher satisfaction, there are relatively few related studies, and the results of the studies are also different due to differences in culture, economic development, and institutional environment. Because teacher satisfaction in developing countries has greater salary flexibility, Meng and Wu (25) applied a questionnaire survey of Chinese elementary education teachers and proved that public service motivation of teachers not only directly affects teacher satisfaction but also positively mediates the positive correlation between salary and teacher satisfaction. In western developed countries, teachers generally have relatively high salaries, social status, and promotion space. Therefore, public service motivation could arouse the altruistic desires of teachers and correct unfair preferences. Choi and Chung (26) found that American teachers often show reduced satisfaction and even leave their jobs due to unfairness in schools and society.

As a member country of the former Soviet Union, Estonia has experienced a series of economic and political reforms and has achieved economic take-off and a leap in the field of education. However, the uneven development of urban and rural education and the problems of education equity caused by history have not been well-resolved. Whether public service motivation can bring excessive incentives to low-income teachers in rural areas has attracted the attention of government policymakers in Estonia and even eastern European countries. However, there is still little research in academia concerning the effect of public service motivation on job satisfaction of teachers, especially in the background of Estonia.

Based on the research objective of exploring the effect of public services motivation on the satisfaction of teachers in the background of urban and rural Estonia, this paper proposes the following hypothesis:

Hypothesis 1: Public service motivation can positively affect the job satisfaction of Estonian teachers.
Hypothesis 2: The influence of public service motivation in rural Estonia on teacher satisfaction is greater than the influence of urban public service motivation.

## Research Method

### Data Source and Variable Design

Because of the demand of scale and coverage (urban and rural) of sample, this article selects the Teacher and Teaching International Survey (TALIS) project database initiated by the Organization for Economic Cooperation and Development (OECD) as the main data source for the research. TALIS has been launched since 2008 for teachers and principals in all OECD countries and some emerging countries. The main content of its teacher part includes background information of teachers, teaching ability, teaching environment, class conditions, etc. The survey is renewed every 5 years. On the aspect of teachers, TALIS was applied to measure teacher's ability, work environment, self-efficacy as well as wellbeing. Moreover, several research applied TALIS to measure teacher's motivations (27), engagement (28) as well as intention to change the school (29), these studies extended the applications of TALIS which also consist of the potential variables of public service motivation. On the specific background of Estonia, TALIS is applied to explore environment's influence on teacher behavior (30), this study finds implications for Estonia education system to improve teacher's innovative teaching skills. Estonia has joined in TALIS since 2008. In the latest round (2018) of the survey, a total of 3,004 questionnaires in Estonia were included in the database, accounting for 20.9% of the total number of secondary school teachers in Estonia.

To compare the different effects of public service motivation, the research needs to divide the teachers participating in the questionnaire into two groups: rural and urban. The basis is question 10 in the interview with the principal of TALIS “What level of city is the school in?” The division of urban and rural areas has formed a unified division standard in economics, sociology and education. This study delineates villages with fewer than 3,000 people as rural areas, including small towns with 3,001 to 15,000 people, cities with 15,001 to 100,000 people, cities with 100,000 to 1 million people, and large cities with more than 1 million people, divided into cities and towns to form a rural-urban dichotomous variable. The explained variable of the study is teacher satisfaction. Question 53 in the TALIS survey covers teacher satisfaction in all aspects, and the last sub question is a summary of job satisfaction. Therefore, the author selects this question as the explained variable. In terms of independent variables, public service motivation is divided into four main dimensions: public service commitment, public policy participation, sympathy, and self-sacrifice. After, most of the previous research concerning about public service motivation use questionnaire to measure this variable and the variable is based on Perry's dimensions on public service motivation. In terms of educational research, despite of the investigation data, some research also applied panel data in different database. For instance, Tao and Wen (31) use Education Panel Survey to measure Chinese teacher's public service motivation, Pedersen (32) applies Danish National Center for Social Research to measure teacher's public service motivation. Because of the plenty of participants and the content of motivations of being a teacher is included in TALIS teacher's questionnaire, the author also applied this database to measure public service motivation of teacher. Vandenabeele et al. (33) and Brewer et al. (34) argued that Perry's four dimensions of public service motivations overlapped each other while being measured. In this way, they reduced the dimensions to three aspects: benefit the group of disadvantages; work for the common goods and contribute to the society; value and awareness of participant to the public policy, while some research applied these new dimensions in measurement (35, 36). Compared to the historical survey of TALIS, TALIS 2018 teacher survey adds a section named teacher's motivation, the analysis plan of TALIS 2018 gives the introduction of this section as the “teacher's motivations to teach” (37), in this section, TALIS divides all the motivation into “personal utility motivations,” “social utility motivation” as well as “perception and value of policy.” Despite of the part of personal utility, the social utility motivation includes the specific question that being a teacher could contribute to the society as well as contribute to the social disadvantages, the section of perception and value of policy contains the teacher's attitudes toward engaging in policy which could reflect motivation from public policy participant. In terms of control variables, the study separately controlled regional variables and individual variables. Among these variables, the regional variable is the degree of poverty in the region that may affect teacher satisfaction. According to the measurement methods in the OECD research report, the author applies the proportion of students with family economic problems in the class to measure the poverty level of the teacher's area (38). In terms of personal variables, the research controls the four aspects of teachers, which include gender, educational background, working years, and work pressure. The names, abbreviations, variable types, and corresponding questionnaire questions of the research variables are shown in Table 1.

**Table 1:** Variables and description.

**Variable**	**Abbreviation**	**Variable type**	**Question**	**Question number**
Rural/urban	T/C	Categorical	Which best describes this school's location?	Principal Q10
Job satisfaction	JS	Explained	All in all, I am satisfied with my job.	Q53j
Contribute to the society	CTS	Independent	Teaching allowed me to provide a contribution to society.	Q7g
Participant of policy	EPC	Independent	Teachers can influence educational policy in this country/region.	Q54d
Benefit social disadvantages	BSD	Independent	Teaching allowed me to benefit the socially disadvantaged.	Q7f
Work experience	WE	Control	Years working as teacher in total.	Q11b
Educational degree	ED	Control	What is the highest level of formal education you have completed?	Q3
Gender	Gen	Control	Are you female or male?	Q1
Work pressure	WS	Control	I experience stress in my work.	Q51a
Poverty degree	PD	Control	Students from socioeconomically disadvantaged home.	Q35e

### Research Method and Model Establishment

Because the explained variable is based on the answer to the questionnaire, it is an ordinal categorical variable, and the independent variable also has multiple categories. Thus, the study selects ordinal regression as the research method to estimate the impact of different public service motivations on teacher satisfaction and the urban-rural differences in impact. The regression model is as follows:


(1) $$\begin{array}{r} \text{Teacher satisfaction }=\;\beta +\beta_{1}\text{PSM}+\beta_{2}\text{Contrl}+\varepsilon \end{array}$$

where the dependent variable is teacher satisfaction, PSM is public service motivation, Control is the control variable, β is a constant value, β1 and β2 are influence coefficients, and ε is an error term. If β1 is positive and the *p*-value is < 0.1, hypothesis 1 can be verified. If β1 of the rural grouping is greater than the β1 of the urban grouping and the *p*-values are all < 0.1, then hypothesis 2 can be verified.

## Results and Discussions

### Descriptive Analysis

This article first divides the research variables into two groups, rural and urban, for descriptive statistics. As shown in Table 2, after removing the missing variable samples, there are 1,173 Estonian rural teacher cases and 1,750 Estonian urban teacher cases in the research TALIS database. In addition, urban teachers face greater pressure than rural teachers, while rural teachers are in a relatively poor regional environment. In terms of overall teacher satisfaction, urban teachers and rural teachers are roughly the same.

**Table 2:** Variable descriptive statistics and comparison between urban and rural areas.

	**Rural group (1,173)**			**Urban group (1,750)**		
**Variable**	**Mean**	**Std**	**Var**	**Mean**	**Std**	**Var**
CTS	3.12	0.84	0.65	3.19	0.821	0.67
BSD	2.77	0.913	0.83	2.75	0.925	0.86
EPC	2.15	0.595	0.354	2.1	0.639	0.41
WE	22.01	13.343	178.04	22.23	12.873	165.72
WS	2.63	0.776	0.60	2.73	0.806	0.65
PD	2.01	0.88	0.78	1.84	0.8	0.64
JS	3.09	0.459	0.21	3.12	0.494	0.24

### Reliability and Validity Test and Correlation Analysis

Because variables such as public service motivation and teacher satisfaction are all derived from the TALIS scale, the research first uses the Cronbach α-coefficient to test reliability. The Cronbach alpha coefficient of the three public service motivation indicators is 0.769, which is higher than 0.7, and the reliability of the scale is relatively high. In the validity test, the KMO fitness measure is 0.724 (>0.7), and the Bartlett sphere test significance is 0.000, which proves that the questionnaire variables can be used for factor analysis. Further measurements show that the factor loads of contribute to the society, benefit the social disadvantages, and participation in policymaking are 0.877, 0.776, and 0.824, respectively, which are all >0.7, indicating that the data have high structural validity.

Before performing regression analysis, it is necessary to analyze the correlation between variables. Table 3 shows the Pearson correlation and significance between the variables: the dual structure of urban and rural areas has largely affected regional economic development, teacher age, gender structure, educational background, and work pressure of teachers while the influence on teacher satisfaction is not significant; both public service motivation variables have a significant positive impact on teacher satisfaction. As a control variable, work pressure will also have a significant negative impact on teacher satisfaction. To further explore the causal relationship between the independent variables and the explained variables and to compare rural cities and towns, the study will adopt ordinal regression.

**Table 3:** Correlation of each variable.

	**T/C**	**JS**	**CTS**	**EPC**	**BSD**	**WE**	**ED**	**Gen**	**WS**	**PD**
T/C	——									
JS	0.03	——								
CTS	0.05*	0.14*	——							
EPC	−0.04*	0.13**	0.15**	——						
BSD	−0.01	0.07**	0.58**	0.1**	——					
WE	0.02	0.01	−0.06	−0.05**	−0.07	——				
ED	0.12**	−0.02	−0.03	−0.05	−0.09*	0.21**	——			
Gen	−0.04*	−0.02	−0.04	0.06**	−0.09**	−015	−0.25	——		
WS	0.06**	−0.22**	−0.06**	−0.17**	−0.05**	0.1**	0.21**	0.09**	——	
PD	−0.1**	−0.05	0.04*	0.01	0.06**	0.11	−0.04*	0.05	0.11**	——

**p < 0.05; *p < 0.1.

### Results

Table 4 shows the influence coefficient and significance of the ordinal regression results. To facilitate the verification of the impact of different public service motivations on teacher satisfaction and the comparison between rural and urban areas, the study divided the research results into three groups based on three different public service motivations, each of which includes rural and urban areas. In terms of control variables, the study found that the satisfaction of male teachers in rural Estonia is significantly lower than the satisfaction of female teachers, while there is no significant gender difference in teacher satisfaction in urban areas, which also reflects the current situation in which most rural teachers in Estonia are female. In addition, the satisfaction of teachers in rural areas of Estonia will decrease significantly with the increase in teaching years, which is also an urgent problem in Estonia where teachers are aging. Moreover, the work pressure perceived by teachers, regardless of urban or rural areas, will have a significant negative impact on teacher satisfaction. The degree of regional poverty has also significantly reduced the satisfaction of rural teachers because the availability of resources for teachers and students in rural schools has increased the difficulty of teaching, thereby affecting teacher satisfaction. Last, in terms of academic qualifications, highly educated teachers in urban areas of Estonia have relatively low satisfaction, possibly because urban teachers with high academic qualifications believe that their talents have not been fully utilized, and academic qualifications have no significant impact on the satisfaction of rural teachers.

**Table 4:** Impact coefficient and urban-rural comparison.

	**Model 1 (CTS)**		**Model 2 (BSD)**		**Model 3 (EPC)**	
**T/C**	**Rural**	**Urban**	**Rural**	**Urban**	**Rural**	**Urban**
GEN	−0.61***	−0.07	−0.6***	−0.07	−0.7***	−0.11
ED	0.08	−0.17**	0.1	−0.17**	0.06	−0.15**
WE	−0.02**	0.01	−0.02**	0.01	−0.02**	0.05
WS	−0.74***	−0.71***	−0.76***	−0.71***	−0.71***	−0.69***
PD	−0.18**	−0.03	−0.16*	−0.02	−0.14*	−0.02
Mid-low PSM	−0.29	−0.07	−0.04	−0.29	0.39	0.29*
Mid-high PSM	0.61*	0.251	0.03	0.18	0.83***	0.51***
High PSM	1.13***	0.66**	0.51**	0.5*	2.75***	1.46***

The control group is low public service motivation group, ***p < 0.01; **p < 0.05; *p <0.1.

Because the statement on public service motivation in the TALIS questionnaire has four scale-based answers ranging from low to high, public service motivation constitutes a non-continuous sequential independent variable. According to the principle of ordinal regression, the study uses the group with the lowest public service motivation as the control group to calculate the parameter estimates and significance of the influence of the middle-low public service motivation, the middle-high public service motivation, and the high public service motivation on teacher satisfaction. In Model 1, both the middle-high and high contribute to society categories in the rural group have a significant positive impact on teacher satisfaction, while in urban areas, only the high contribute to society group has a significant positive impact, and the impact coefficient is also obvious. The public service motivation variable of Model 2 is benefit social disadvantages. In Table 4, benefit social disadvantages shows a positive effect on teacher satisfaction only in the high public service motivation group compared with the low public service motivation group. There is no significant difference in the coefficient of influence between rural and urban areas. Finally, the motivation to participate in education policy has a significant impact on teacher satisfaction in Estonia, and it also shows that the effect of rural areas is greater than that of urban areas, which is similar to the results of Hulpia et al. (18). In general, Model 1 and Model 3 tend to accept Hypothesis 1 and Hypothesis 2. Model 2 validates Hypothesis 1 but rejects Hypothesis 2. Generally, the hypothesis in the second part of the study is verified; that is, public service motivation can positively affect teacher satisfaction, and rural areas have a higher marginal effect than urban areas^2^.

### Discussion of Research Results

The phenomenon of the urban-rural dual structure mentioned in the paper appears not only in the path coefficients affecting public service motivation but also in demographic and environmental variables. For countries with a large gap between urban and rural areas, the salary of rural teachers is not only lower than the salary of urban teachers but also lower than the average salary of other occupations in rural areas. Due to factors such as the family division of labor, the satisfaction of male roles is more flexible than the satisfaction of female roles. As a result, male rural teachers in Estonia are less satisfied, while female rural teachers are relatively more likely to accept the profession of teachers. At the same time, research has proven that rural teachers in Estonia show a decreasing trend of satisfaction with age. As teachers grow older, the demand for public services increases, including sports, medical care, community services, etc. The dual structure of urban and rural areas indicates that the accessibility of public services for rural teachers is significantly behind the accessibility of public services for urban teachers; at the same time, as rural teachers age, the living conditions and education problems of their children have become important factors affecting their happiness. Therefore, these problems are important reasons for the decline in the satisfaction of teachers in rural Estonia with age. In addition, the poverty level of the school area has a greater impact on the satisfaction of rural teachers. On the one hand, the economic problems of families of students will spend more energy on teachers, thereby reducing the satisfaction of teachers; on the other hand, it also illustrates the duality of urban and rural education in Estonia, and rural teachers in poor areas face excessive work pressure and single-mindedness. With the performance appraisal model and less extracurricular training, teacher satisfaction will decrease significantly.

In terms of public service motivations, the urban teacher's impact coefficient of public policy participation and motivation of contribute to society is significantly smaller than the impact coefficient of rural teachers. There are three main reasons for this: first, there are many other factors that affect teacher satisfaction in urban areas (salary, promotion opportunities, training, awards), and the process of various factors acting on teacher satisfaction is prone to “crowding out,” while teacher education resources in rural areas are relatively scarce and resource accessibility for teachers is quite low, so public service motivations have a higher marginal effect in affecting teacher service satisfaction. Second, while public service motivation directly affects teacher satisfaction, it can also mediate the impact of other factors on teacher satisfaction. Factors such as low salary, low training opportunities, and low support for teaching activities of rural teachers significantly negatively affect teacher satisfaction, and the high public service motivation of teachers can reduce these negative effects to a certain extent. Public service motivation has much more mediating effects in rural areas, so the overall impact is greater than in urban areas. The third is based on the specific situation of the Estonian country. Due to the migration of the rural population to the city and the continuous decrease in the population growth rate, the teacher-student ratio in rural schools has dropped significantly. In this case, a variety of working mode options (part-time, distance learning), gradually reduced workload and job stability make it easier for rural teachers to be satisfied. After all, the public service motivation in Estonia has made a great contribution to the satisfaction of domestic teachers, also showing that teacher satisfaction is more affected by internal factors rather than external factors, and the internal factors can also compensate for external factors to a certain extent (39).

### Implications and Educational Strategy of Estonia

Estonia is a former Soviet Union country with high ethnic heterogeneity. The number of Russians in the country increased from 8% in 1939 to 35% in 1989, and 32% of non-Estonian people remain in the country (40). Under the influence of economic and political reforms, Estonia has experienced educational reforms since the founding of the state with three main orientations: decentralization, depoliticization, and democratization. According to the Economist's Intelligence Unit report, the white paper on democratic indicators (41), the degree of democracy in Shania received an overall score of 7.9, ranking first in Eastern Europe. High democratic scores and the sinking of power have greatly reduced the transmission time of education policies and increased the efficiency of education policies. In terms of Estonia's teacher policy, on the one hand, the quality of teachers is improved through nationalization, and the opportunities for vocational training and studying abroad for teachers are increased to realize cognitive ability and diversification ability of teachers. On the other hand, the relatively disadvantaged group of teachers is carried out. The most typical policy is to provide rural teachers with a one-off subsidy of 12,750 euros, which makes teachers in poor areas more satisfied (42).

The 2020 Estonian Lifelong Learning Strategy specifically points out the focus of Estonia's future teacher policy, which aims to improve teacher satisfaction and increase the accuracy and effectiveness of incentives (43). To complete the new development goals of basic education, the first is to gradually improve the social status and basic salary of teachers to make employment in school the best employment option in Estonia and to ensure the quality of new teachers through a competition model. The second is to attract young and male teachers, gradually achieve a structural balance of the teaching team, and increase the number of young teachers in rural schools. The third is to improve the performance-salary incentive mechanism, set up a teacher self-assessment system to record the evolution of cognitive ability of teachers and set up a principal association to regularly discuss teacher performance evaluation and rewards and punishments. The fourth is to increase the level of autonomy of local schools and allow a greater number of teachers to participate in school decision-making and rules and regulations. At the same time, it will also open up channels between teachers and education policy makers and enhance motivation of teachers for public policies.

## Conclusion

To explain the high level of teacher satisfaction in Estonia, this article explores the origin of Estonian educational achievements from the perspective of the urban-rural dual structure with the perspective of public service motivation and teacher satisfaction. Through the quantitative analysis of TALIS data, the study found that the public service motivation of Estonian teachers significantly promoted the improvement of teacher satisfaction, and the effect in rural areas was more significant. This article has two limitations. One is that TALIS's indicator of teacher satisfaction and public service motivation is a four-point scale, which affects the accuracy of the variables to a certain extent; the other is that there are relatively few problems in measuring public service motivation. To explore the multipath mechanism of multidimensional public service motivation to teacher satisfaction or to change the research object, future research can use in-depth interviews and structural equation methods to further explore this issue on the basis of reducing the sample size.

## Data Availability Statement

The original contributions presented in the study are included in the article/supplementary files, further inquiries can be directed to the corresponding author.

## Author Contributions

The author confirms being the sole contributor of this work and has approved it for publication.

## Funding

This dissertation was funded by the One Belt One Road National and Regional Education System Research, a major project of the China Ministry of Education's Philosophy and Social Science Research (project number: 19JZD052).

## Conflict of Interest

The author declares that the research was conducted in the absence of any commercial or financial relationships that could be construed as a potential conflict of interest.

## Publisher's Note

All claims expressed in this article are solely those of the authors and do not necessarily represent those of their affiliated organizations, or those of the publisher, the editors and the reviewers. Any product that may be evaluated in this article, or claim that may be made by its manufacturer, is not guaranteed or endorsed by the publisher.

## Footnotes

^1^Teacher Teaching International Survey (TALIS) is the only existing database enable the author to explore the status of teacher's satisfaction and different dimensions of public service motivation in Estonia.

^2^The author conducts the robustness check to test the external validity, firstly the author exchanges the data as the TALIS data in 2015 and the significance remain the same; secondly the author changes the method of multiple logistic regression to examine the robustness of ordinal regression in this article, the significance of different public service motivation and the higher effect in rural Estonia is also get the same result as above which proves a good robustness.
